# WHODAS Assessment Feasibility and Mental Health Impact on Functional Disability in Systemic Lupus Erythematosus

**DOI:** 10.3390/healthcare10061053

**Published:** 2022-06-06

**Authors:** Liliana Duca, Nadinne Alexandra Roman, Aliana Miron, Andreea Teodorescu, Lorena Dima, Petru Ifteni

**Affiliations:** 1 Faculty of Medicine, Transilvania University of Braşov, 500036 Braşov, Romania; liliduca@yahoo.com (L.D.); aliana_mioc@yahoo.com (A.M.); andreea.teodorescu@unitbv.ro (A.T.); lorenadima@yahoo.com (L.D.); petru_ifteni@yahoo.com (P.I.); 2 Department of Clinical Immunology, County Emergency Clinic Hospital, 500326 Braşov, Romania; 3 Clinical Hospital of Psychiatry and Neurology of Brasov, 500123 Braşov, Romania

**Keywords:** systemic lupus erythematosus, depression, anxiety, disability, WHODAS

## Abstract

Systemic lupus erythematosus (SLE), besides rheumatological dysfunction, manifests in neuropsychiatric disorders like depression and anxiety. Mental health illnesses in SLE patients have a high prevalence and a profound impact on quality of life, generating an increased disability and premature mortality. This study aimed to establish the degree of disability in patients with SLE and the impact of depression and anxiety on patients’ functioning. Additionally, the study aimed to verify whether World Health Organization-Disability Assessment Schedule (WHODAS) 2.0 is suitable for the evaluation of patients with SLE associating depression and/or anxiety symptoms. Cross-sectional research was performed, including adult patients, diagnosed with SLE. To evaluate depression, anxiety, and functioning, approved questionnaires Hamilton Anxiety Rating Scale, Hamilton Depression Rating Scale, and, World Health Organization-Disability Assessment Schedule (WHODAS) were applied. Confirmatory factor analysis was performed on WHODAS subscales. Sixty-two patients were included in the research, with a mean of SLE diagnosis of 12.48 years; 53 patients (85%) had depression (*p* < 0.001). Anxiety was found in 38 patients (61.29%, *p* < 0.05). WHODAS assessment results depicted that 39 patients (62.90%, *p* < 0.05) manifested disability, from which 26 (66.66%, *p* < 0.05) presented moderate and severe disability. A strong correlation between the severity of anxiety and the degree of disability (r > 0.6, *p* < 0.001) was found. The WHODAS scale assessment proved to be a valuable tool for SLE patient’s functioning assessment. This study suggests that depression and anxiety negatively impact WHODAS disability scores, decreasing the quality of life in SLE patients.

## 1. Introduction

Systemic lupus erythematosus (SLE) is a clinically heterogeneous autoimmune disease and with complex pathogenesis. As the diagnosis of the disease improved, the incidence almost tripled in the last 40 years of the twentieth century [1]. SLE is widespread in all geographical areas of the world, but despite the increase in survival rate, 15–20% of patients with SLE die within 15 years of diagnosis. According to estimates, 500,000 people in Europe have lupus, and worldwide the data indicate that 5 million patients are affected by various forms of the disease. The most common and severe form of lupus, which affects 70% of patients, is systemic SLE, which can affect any system or organ of the body [2]. The etiology of SLE is unknown and currently, no treatment has been developed; only symptomatically medication is administered. Patients with SLE may have complex clinical conditions. they often report pain, fatigue, arthritis, and rashes, that affect the musculoskeletal system and mental health. Other internal organs are affected in the advanced stages of the disease [3]. Because the therapy is predominantly symptomatic, it involves the administration of immunosuppressants, which cause various long-term side effects. Thus, the quality of life (QoL) related to health deteriorates in ratio to the general population, comparable to that of patients with other chronic diseases, which is lower than in the general population. [4]. Regarding the prevalence of anxiety and depression in SLE patients, in Europe, Arnaud et al. found a percent of 30.5% with anxiety, and depression was 15.3% [5]. Furthermore, the COVID-19 pandemic has negatively influenced the quality of life, significantly impacting the patients’ mental health with SLE [6].

Neuropsychiatric lupus (NPSL), as a form of manifestation of SLE, is the least understood in terms of pathology; it is also perhaps the most prevalent manifestation of the disease. It affects 14% to 80% of adults and may occur independently of the disease’s activity [7].

Among the psychiatric manifestations, depression and anxiety have a high prevalence in patients with SLE. Current epidemiological data indicate variable percentages ranging from 8.7–78.6% and 1.1–71.4%, respectively, depending on the scales used and the definition of depression and anxiety [8]. Depression and anxiety profoundly impact the quality of life; they are associated with an increased suicide risk, premature mortality, and increased disability [9,10]. Thus, early detection and proper treatment of these disorders are essential elements in the goal-setting of medication regarding the drugs’ psychiatric side effects association and concerning goals settings allied to mental health and locomotor function of patients with SLE. Hence, a depression, anxiety, and daily functional assessment screening can improve the evolution and QoL in patients with SLE [11].

Studies performed on patients with SLE indicated the negative impact that anxiety and depression have on the evolution of lupus and the disease-related costs [12,13]. The functional deficits associated with depression are long-lasting [14]. They may exceed the severity of those associated with other chronic diseases such as diabetes mellitus, high blood pressure, and heart failure [15]. Anxiety and depression are identified as factors associated with social and individual difficulties, including high health care costs, and an increased risk of physical comorbidities, such as cardiovascular disease, also have been linked to poor QoL in much research [16]. The locomotory system is also altered, hence converging the mental issues, with poor QoL level and the inability of self-care, determining a tremendous burden on mental and physical disability [17].

Among patients who meet the criteria for major depressive disorder, almost 60% have a very severe functioning deficit [18] impacting different areas such as domestic activities, work, friends, and family, seriously altering the capacity for self-care and autonomy [19].

To assess the degree of disability, the World Health Organization-Disability Assessment Schedule (WHODAS 2.0) suggests using it as a tool with a unique potential to determine the level of functioning of an individual, regardless of the pathology type [20]. WHODAS 2.0 comes bundled with the Diagnostic and Statistical Manual of Mental Disorders (Fifth Edition, DSM-5), and is endorsed as a new and valuable measure of functional impairment in psychiatric disorders [21].

This study aimed to establish the degree of disability in patients with SLE and the impact of depression and anxiety on patients’ functioning. Additionally, the study aimed to verify whether WHODAS 2.0 is suitable for evaluating patients with SLE associating depression and/or anxiety symptoms.

## 2. Materials and Methods

### 2.1. Participants

A cross-sectional study, including 62 adult outpatients, diagnosed with SLE according to SLICC or ACR criteria for at least 6 months before the enrollment, was included in the research. The patients were recruited between June 2019–January 2020 [22,23]. The study was conducted in the Department of Clinical Immunology, County Emergency Clinic Hospital of Braşov, Romania. The local ethical committee approved the research. Also, all patients provided written informed consent to participate in the study. All procedures were conducted according to the local regulations. The disease duration was evaluated at the time of meeting the diagnostic criteria for SLE.

One of the research exclusion criteria was no previous NPSL diagnosis. Therefore, the patients enrolled in the research should have received no medication or therapy for anxiety or depression disorder. All patients with a history of substance abuse, personality disorder, or other major psychiatric disorders were excluded. Patients who met the criteria for alcohol abuse were omitted.

Demographic data were collected from all patients, including age, gender, education, employment status (active/inactive/retired), marital status, smoking, and alcohol consumption.

Patients underwent a complex clinical evaluation, including a complete physical and biological examination with serological determinations for SLE. The assessment also investigated inflammation status, complete blood count (CBC), renal function, complement C3 and C4 level, antinuclear antibody profile (Ab anti -dsDNA, anti-Sm, anti- Ro, anti-La, anti-histone, anti-RNP, anti-ribosomal P protein), coagulation tests.

The instruments used to evaluate SLE activity were the British Isles Disease Activity Group Index 2004 (BILAG Index) and the Systemic Lupus Erythematosus Activity Index (SELENA-SLEDAI). Only patients with no disease activity were included.

### 2.2. Outcomes

WHODAS 2.0 measures average functioning in everyday situations for the last 30 days and surveys six domains of functioning: (1) cognition (understanding and communicating), (2) mobility (ability to move and get around), (3) self-care (e.g., about hygiene, dressing, and eating) (4) getting along with others, (5) life activities (ability to attend to everyday responsibilities), and (6) participation in society [20]. The scores assigned to each item are recorded and summed in each domain with a range from 0 (best) to 100 (worst) [20].

In order to analyze the disability degree, thresholds based on ICF International Classification of Functioning qualifying percentages were used: absent (0–4%), mild (5–24%), moderate (25–49%), severe (50–95%) and extreme (96–100%). (16). The most widespread and evaluated form of the WHODAS 2.0 is the 36-item structured interview version, which takes approximately 20 min to complete and has excellent psychometric properties [20].

Patients were evaluated for levels of depression and anxiety by a certified psychiatrist using Hamilton Anxiety Rating Scale (HAM-A) and the 17-item Hamilton Depression Rating Scale (HAM-D17) [24,25]. Depression was defined at a HAM-D score of 8 or more (8–17 mild depression, 18–25 moderate depression, >26 severe depression). Anxiety was defined at a HAM-A score of 8 or more (<7 absent anxiety, 8–14 mild anxiety, 15–23 moderate, and ≥24 severe anxiety) [24,26]. HAM-A and HAM-D proved to be short, reliable, and valid tools for mental health assessment. [27,28,29].

The degree of disability was assessed using WHODAS 2.0. [30,31]. The WHODAS assessment tool proved to have good psychometric properties and also suitable reliability and validity [32]. The WHODAS questionnaire was applied in the Romanian language, using the form provided by a professional psychology association [33]. HAM-D and HAM-A were also used in Romanian, in the translated and adapted form, using a tool provided by Romanian Psychological Testing Services [34].

### 2.3. Statistical Analysis

All collected data were analyzed using Statistical Package for the Social Sciences (IBM SPSS Statistics for Windows, Version 20.0. Armonk, NY, USA: IBM Corp.). We performed the structural equation modeling (SEM) using Amos (Version 20.0), Chicago: IBM SPSS. The Pearson correlation coefficient was determined for pairs of studied variables. The correlation coefficients and the p-values were calculated according to a default 95% confidence interval. The significance level was set at p values less or equal to 0.05. The Chi-square test was used for univariate comparison between categorical variables. We used a nonparametric test, since data were not normally distributed, while the One-Sample Kolmogorov-Smirnov Test was used for continuous variables. Binary logistic regression was used to detect possible risk factors for depression and anxiety.

A post hoc power, sample, and effect size were computed using G * Power (latest version 3.1.9.7; Heinrich-Heine-Universität Düsseldorf, Düsseldorf, Germany) for the 62 participants sample size. For α = 0.05, the power was 0.997 (1–β err prob), while the effect size was 0.707, suggesting a medium effect size.

To identify if the WHODAS instrument is valid and identify the measured variables, we performed an exploratory factor analysis (EFA). We applied Principal Axis Factoring and Varimax rotation with Kaiser Normalization as the extraction method, and the assumptions of linearity and correlation were verified. All variables should correlate with at least one other variable, with r ≥ 0.3. Kaiser–Meyer–Olkin (KMO) was investigated for sampling suitability also the Bartlett sphericity test. We considered the following values of the KMO > 0.5 and Bartlett *p* < 0.05 as appropriate values for EFA.

After the EFA, a confirmatory factor analysis (CFA) was accomplished using structural equation modeling (SEM) [35]. The following parameters were taken into consideration regarding the model fit: Root Mean Square Residual (RMR) < 0.08, Goodness-of-Fit Index (GFI) ≥0.95, Adjusted Goodness-of-Fit Index (AGFI) ≥ 0.95 [35]. Cronbach’s Alpha value was used to identify internal consistency. We used the Pearson correlation with HAM-D and HAM-A.

As regards the multiple regression analysis, a stepwise procedure was used.

## 3. Results

In Table 1 are found the main group characteristics for continuous and ordinal variables.

The studied population included 62 Caucasian lupus, whose disease was controlled on background therapy. The average duration of the disease was 12.48 years. The distribution by sex was four men (6.45%) and 58 women (93.55%). The mean age was 51.27 ± 13.85 years. Fifteen patients (24.19%) were smokers.

From 62 participants, 5 (8.06%) were widowed, 35 (56.45%) married, 8 (12.90%) unmarried, while 14 (22.58%) were divorced. As regards the social status, 6 (9.68%) were unemployed, 33 (53.23%) were retired, and 23 (37.10%) participants declared were an employee. The applied questionnaires’ leading disability, depression, and anxiety correlations are found in Table 2.

Severe depression was reported in 7 patients (11.29%), 21 (33.87%) patients had moderate depression, while 25 (40.32%) reported mild depression, and nine (14.52%) participants had no mental disorder related to depression. As regards anxiety, just one patient (1.61%) confirmed that no anxiety was present. In contrast, 15 (24.19%) reported very severe anxiety, six participants (9.68%) accounted for severe anxiety, nine patients (14.52%) declared moderate anxiety, while 31 (50%) were recorded with mild anxiety.

HAM-A chi-square test results suggest a statistically significant difference regarding the anxiety level in the assessed population, with *p* < 0.001, R^2^ = 43.161 (4 degrees of freedom), depicting that mild anxiety exceeded more than double the expected value for the equal proportion of the anxiety levels. Regarding HAM-D results for the Chi-Square test, mild and moderate depression exceeded the expected value, while none and severe depression categories accounted for less than the expected value, R^2^ = 15.161 (2 degrees of freedom), and *p* = 0.002.

The WHODAS assessment results showed that 13 patients (20.97%) were not disabled. In contrast, 44 (70,97%) had mild disability, and 5 (8.06%) had moderate disability, with R^2^ = 41.065 and *p* < 0.001 (2 degrees of freedom), overcounting the number of participants with mild disability.

WHODAS assessment showed that the most affected areas were participation in society (45.76% of patients) and daily activities related to home/service/school care (44.96% of patients). Patients also reported some degree of disability related to interpersonal relationships (32.98%) and mobility (32.41%). Impairment of cognition was found in 20.69% of patients and impairment of self-care in 15.42% of patients.

We have analyzed the statistical significance between the time since SLE onset and depression and anxiety assessment using Kruskal Wallis Test. The comparison showed no significant differences.

Regarding correlation values and statistical significance, all WHODAS subscales correlated above 0.622 (*p* < 0.001) with HAM-A and HAM-D final scores, and each other; the results can be seen in Table 3.

**Figure 1:**
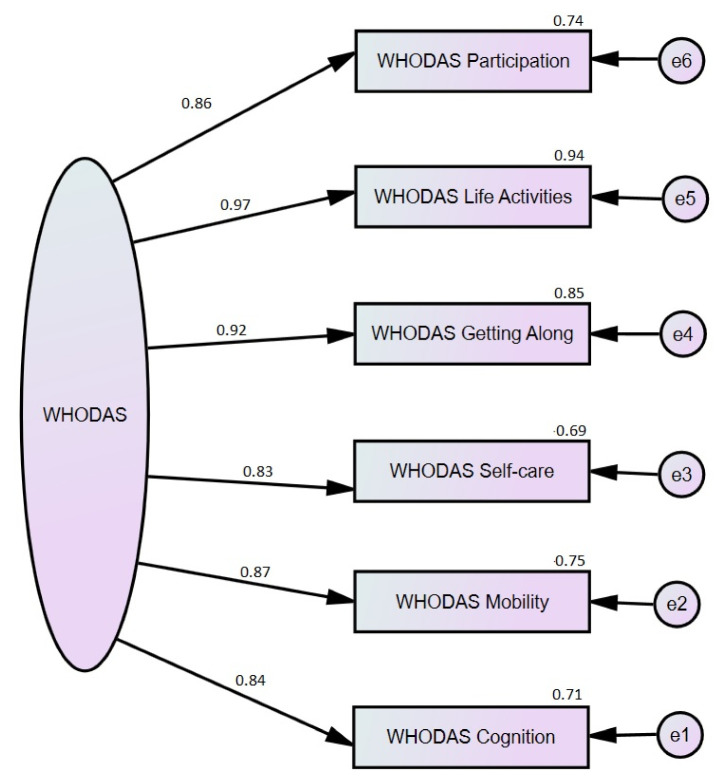
CFA diagram on SEM for the WHODAS scale assessment. (e1 to e6 are endogenous, unobserved variables).

As regards the other variables analyzed for correlation, the educational level was negatively correlated with all WHODAS subscales, with R between 0.497 and 0.660, and HAM-D and HAM-A scores, R = 0.470, respectively 0.597, *p* < 0.001. The duration of time since patients have had SLE was also statistically significant correlated with WHODAS subscales (values between 0.251 and 0.521), with the strongest correlation with the Mobility and Self-care subscales suggesting moderate correlations. To determine if the WHODAS instrument used in our research is a valid tool to detect functional disability among LES patients, EFA and CFA were performed. The results are depicted in Table 4 and Figure 1.

The KMO value for the EFA was 0.876, and Chi-Square for Bartlett’s test of sphericity was 393.88. The EFA results on the variance explained depicted 1 factor with 4.79 Eigenvalue, explaining 76.20 of the total variance.

Figure 1 schematically shows the CFA result. Regarding the reference indices, RMR = 0.078, GFI = 0.997, AGFI = 0.994, while the minimum model fit was achieved. Regarding the linear regression results, the statistical analysis was performed considering HAM-D, HAM-A, and WHODAS total scores as independent variables. Also, to identify the reliability, the Cronbach Alpha was computed on WHODAS subscales, resulting in a value of 0.952, suggesting a high liability. The results of linear regression are shown in Table 5.

The linear regression results for HAM-D revealed two models in which anxiety (HAM-A) and WHODAS Participation influence the degree of depression (HAM-D). As regards anxiety (HAM-A), the linear regression results suggest that firstly, depression (HAM-D) influence anxiety, altogether with the educational level, in harmful matter (as lower the education level is, the depression severity increase), and also combined with total WHODAS score. The functional disability linear regression results suggest five models that explain the influence of various elements on the WHODAS total score. As shown in Table 5, firstly, depression and secondly, increased age influence functional disability. The fifth model encounters five elements: depression, increased age, use of cortisol medication, the length of SLE disease, and gender (more predominant in females) influence in a substantial proportion of the WHODAS score, considering the value of R^2^ (0.935), and is statistically significant.

## 4. Discussion

In our study, based on HAM-D17 and HAM-A, rates of depression and anxiety in SLE patients were 85% and 61.29%, respectively, which are higher than previously reported. A similar study found a 45.2% rate for depression and a 37.1% rate for anxiety [36]. Sex distribution in the study group was 93.55% female and 6.45% male, which is similar to the existing data. SLE usually has a female/male sex ratio of 9-10/1 [37]. A percent of 48.38% of the patients in our study group reported both depression and anxiety symptoms. An association between depression and anxiety was also noted by previous studies [36]. Although recent studies indicate that depression in lupus might be a side effect of corticosteroid therapy, reducing the brain-derived neurotrophic factor [3], our study showed no correlation. However, the patients involved in our research only included therapeutically controlled lupus disease. Corticosteroids for the studied subjects’ doses were lower than 10 mg Prednisone or equivalent (dose considered at risk for the occurrence of depression). The patients had stable doses in the last four weeks. Hence, the linear regression results may explain why it did not prove that cortisol medication influences disability, anxiety, or depression in SLE patients [37].

An interesting correlation was found between depression and educational level: in our study, a higher level of education was correlated with an increased risk of depression (*p* < 0.05). In the general population, a lower educational status that decreases access to employment opportunities is associated with an increased risk of depressive symptoms [38,39]. SLE patients’ previous studies found that unemployment and lower education levels are associated with depression [32,40]. However, our study shows that a higher education level correlates with an increased risk of depression, while employee status does not influence depression rates. Also, we found that the marital status did not influence the depression scores in the SLE patients, even though existing data shows a clear correlation between the two [41].

In this group of SLE patients, those under 50 seemed to be at a higher risk for anxiety. The correlation we found between anxiety and age is consistent with existing data. In the general population, the age at the onset of generalized anxiety disorder is between 21.1 and 34.9 years [42]. Regarding the specific population of SLE patients, Maneeton et al. found that anxiety and younger age are associated with depression [43]. The correlations found between anxiety scores and education level or employment status show that a higher level of education and being employed have a protective role against anxiety. Similarly, previous studies suggest that a higher anxiety level is associated with lower education and lower income. At the same time, a better quality of life was significantly related to lower stress/anxiety/depression, higher education, and higher income [44,45]. Marital status is a significant predictor of perceived stress, specifically the stresses associated with social commitments, loneliness, and economy/money. These domain-specific stressors also mediated the relationship between marital status and anxiety [46]. However, in this study, marital status does not appear to impact anxiety scores.

A complete and complex approach to the SLE patient should use a battery of tools that include: assessment of fatigue, pain, depression, anxiety, general health, and quality of life scores that will be correlated with the clinical and biochemical evaluation. Clinicians currently have at their disposal validated tools for lupus disease that have shown their utility on economic models: SLE-specific health-related quality of life (HRQoL), The Functional Assessment of Chronic Illness Therapy-Fatigue Scale (FACIT-Fatigue), Brief Pain Inventory (BPI-SF), The Hospital Anxiety and Depression Scale (HADS), Short Form [36 items] Health Survey version 2 (SF-36v2), EuroQoL 5-dimensions (EQ-5D-5L) and Work Productivity and Activity Odd Questionnaire: Lupus (WPAI: Lupus) [11].

Aside from the punctual evaluation of specific lupus symptoms, establishing the functional impact that depression and anxiety symptoms have on the SLE patient is essential. In this regard, WHODAS can provide a global assessment of the functional level and the disability degree. A 2017 meta-analysis shows that WHODAS 2.0 is suitable for evaluating patients from different populations and with various pathologies [47]. WHODAS 2.0 scores are significantly different in mild versus severe depression [48]. Moreover, the scale appears to classify the illness’ severity correctly. It identifies item 6.6 (effect of disease on personal finances) and item 4.5 (impairment of sexual life) as the items that best correlate with disease severity [49]. We, therefore, considered WHODAS as an appropriate tool for assessing the degree of disability associated with depressive and anxiety symptoms in SLE patients.

The confirmatory factor analysis results on the WHODAS assessment scale used in our research suggest, as previous results performed on different pathologies, that WHODAS is a valuable tool for functional disability evaluation. Our research suggests that WHODAS is feasible and usable in SLE patients, thus becoming a necessary tool for SLE patients’ disability screening and monitoring, and can serve as a method of identifying physical therapy prophylaxis protocols with an impact on both locomotor disability and favoring mental health improvement. [50].

This study revealed a very strong correlation between depression symptoms and severe disability in social participation, interpersonal relationships, and life abilities. In the other areas, patients reported a moderate disability; results in contrast to recent data showing a correlation between depression and moderate disability [51].

There are some limitations to this study. Firstly, there was a small sample size, and therefore, the results should be interpreted cautiously. Secondly, given the higher prevalence of SLE in women, nearly all participants in this study were female, so cautiously, a generalization of these results to male SLE patients should be made. The disadvantages of our small sample research are related to the amount of information provided by the results. Therefore, the estimates may not be as accurate and may be less representative for patients with SLE suffering from anxiety, depression, or both than a larger sample size. Another limitation of this research is the lack of a control group. Although no intervention has been applied and the results are based only on evaluations, future research should identify the level of dysfunction in patients with SLE and impaired mental health. The research should compare SLE patients who receive or do not receive medication or psychological therapy. However previous research has shown similar results in lack of correlation or association. Another limitation of our research concerns the analysis of comorbidities in patients with SLE and the relationship with anxiety, depression, or disabilities further research should include patients at the national level in the study, the presence and type of comorbidities, the prevalence and incidence of depression, anxiety, locomotor disabilities, QoL, and steroid medication.

There are no published data regarding the correlations between depression and anxiety symptoms in SLE and disability in the major functioning areas. Further studies regarding the translation, adaptation, and cultural validation of the WHODAS scale in Romania could potentially validate WHODAS 2.0 as an instrument for evaluating the six major areas of functioning in SLE. Also, since physical and mental disability burden SLE patients, future research, protocols, or guidelines regarding physical and mental health could increase QoL and SLE patients’ independence.

## 5. Conclusions

This study reveals a high prevalence of depression and anxiety in patients with SLE. Therefore, we support the active screening for these symptoms in this population. There is a robust positive correlation between depression and overall functioning disability as shown by WHODAS 2.0 evaluation. WHODAS might be a relevant tool for assessing the disability associated with depression and anxiety symptoms in SLE patients.

## Figures and Tables

**Table 1:** Descriptive Statistic.

Characteristics (*n* = 62)	Minimum	Maximum	Mean/SD	95% Confidence Interval for Mean	
				Lower Bound	Upper Bound
Age	22	77	51.27/13.92	47.74	54.81
Education Level *	1.00	5.00	3.40/1.51	3.01	3.78
Years of smoking history	0	20	2.82/5.25	1.49	4.16
Years of LES	1	38	12.48/8.15	10.41	14.56
Cortisol medication **	0	10	2.77/3.03	2.00	3.54
HAM-D	1	34	16.23/7.94	14.21	18.24
HAM-A	0	3.14	1.36/0.86	1.14	1.58
WHODAS TOTAL	1.88	70.56	32.02/16.09	27.93	
WHODAS Cognition	0	58.33	20.69/17.30	16.30	25.09
WHODAS Mobility	5	70	32.42/16.11	28.33	36.51
WHODAS Self-care	0	62.50	15.42/14.25	11.80	19.04
WHODAS Getting Along	0	70	32.98/18.65	28.25	37.72
WHODAS Life Activities	0	90.63	44.96/21.23	39.56	50.35
WHODAS Participation	6.25	78.13	45.76/19.12	40.91	50.62

* Educational levels were set according to ISCED classification: ISCED 2 Secondary school (gymnasium secondary level), ISCED 3 Upper secondary education (high school education), ISCED 4 Post-secondary non-tertiary education (professional and technical education), ISCED 5 Short-cycle tertiary education (professional college), ISCED 6 Bachelor’s or equivalent level; ** measured in milligrams.

**Table 2:** Assessment’s scoring and Pearson Correlation.

Characteristics (*n* = 62)	MiniMum	MaxiMum	Mean/SD	95% Confidence Interval for Mean		Pearson Correlation	
				Lower Bound	Upper Bound	Value	Sig (*p*)
HAM-D 17	1	34	16.23/7.94	14.21	18.24	0.815 *	<0.001
HAM-A	0.000	3.140	1.36/0.86	1.14	1.58	0.802 **	<0.001
WHODAS TOTAL	1.88	70.56	32.02/16.09	27.93	36.12	0.835 ***	<0.001

* HAM D & HAM A; ** HAM A & WHODAS; *** HAM D& WHODAS.

**Table 3:** Pearson correlation of HAM D, HAM A, and WHODAS total score and sub scores (*n* = 62).

	HAM-D 17	HAM-A	WHODAS Cognition	WHODAS Mobility	WHODAS Self-Care	WHODAS Getting Along	WHODAS Life Activities	WHODAS Participation
HAM-D 17	1	0.815 **	0.753 **	0.625 **	0.622 **	0.820 **	0.814 **	0.839 **
HAM-A	0.815 **	1	0.750 **	0.671 **	0.668 **	0.754 **	0.761 **	0.725 **
WHODAS Cognition	0.753 **	0.750 **	1	0.717 **	0.741 **	0.782 **	0.816 **	0.680 **
WHODAS Mobility	0.625 **	0.671 **	0.717 **	1	0.872 **	0.780 **	0.834 **	0.686 **
WHODAS Self-care	0.622 **	0.668 **	0.741 **	0.872 **	1	0.745 **	0.797 **	0.605 **
WHODAS Getting Along	0.820 **	0.754 **	0.782 **	0.780 **	0.745 **	1	0.886 **	0.852 **
WHODAS Life Activities	0.814 **	0.761 **	0.816 **	0.834 **	0.797 **	0.886 **	1	0.854 **
WHODAS Participation	0.839 **	0.725 **	0.680 **	0.686 **	0.605 **	0.852 **	0.854 **	1

** Correlation is significant at the 0.01 level (2-tailed).

**Table 4:** Factor loadings for WHODAS assessment with EFA and CFA.

WHODAS Subscale	Mean ± SD (*n* = 62)	Communalities Extraction (EFA)	Factor Matrix Loading (EFA)	Standardized Regression Weights SEM (CFA)
Cognition	22.39/16.17	0.601	0.775	0.836
Mobility	32.6/15.91	0.764	0.874	0.863
Self-Care	15.42/14.26	0.718	0.847	0.832
Getting Along	32.98/18.65	0.863	0.929	0.923
Life Activities	44.96/21.24	0.930	0.964	0.973
Participation	45.77/19.12	0.696	0.834	0.856

**Table 5:** Linear regression results on factors that influence depression, anxiety, and functional disability in LES patients.

Model		Unstandardized Coefficients (B)	Standardized Coefficient β	*p*	R^2^
HAM D	WHODAS Participation	0.349	0.839	0.000	0.839
	WHODAS Participation+ HAM A	0.218	0.524	0.000	0.891
		3.991	0.435	0.000	
HAM A	HAM D	0.089	0.815	0.002	0.815
	HAM D+ Educational level	0.075	0.685	0.000	0.851
		−0.158	−0.278	0.001	
	HAM D+ Educational level+ WHODAS Self Care	0.065	0.593	0.002	0.862
		−0.130	−0.228	0.005	
		0.011	0.185	0.043	
WHODAS total score	HAM D	1.691	0.835	0.000	0.835
	HAM D+ AGE	1.531	0.756	0.000	0.898
		0.391	0.339	0.000	
	HAM D+ AGE+ Years of LES	1.560	0.770	0.000	0.915
		0.278	0.241	0.000	
		0.394	0.200	0.002	
	HAM D+ AGE+ Years of LES+ Gender	1.577	0.778	0.000	0.922
		0.289	0.250	0.000	
		0.375	0.190	0.002	
		−7.840	−0.121	0.023	

## Data Availability

Data available on request.
